# The mini-incision donor nephrectomy is best suited for Indian patients undergoing live donor nephrectomy: Against the motion

**DOI:** 10.4103/0970-1591.60465

**Published:** 2010

**Authors:** Pranjal Modi

**Affiliations:** Department of Urology and Transplantation Surgery, Institute of Kidney Diseases and Research Centre, Institute of Trans plantation Sciences, Civil Hospital Campus, Asarwa, Ahmedabad - 380 016, Gujarat, India

## INTRODUCTION

Donor nephrectomy is performed in a healthy human who otherwise does not require any surgery. Between 1954 and 1994, the open donor nephrectomy (ODN) through flank incision was considered as a standard procedure for kidney donation. Flank pain, incisional hernia, neuralgia, and muscle weakness were observed in large number of donors undergoing ODN. Ratner performed the first laparoscopic donor nephrectomy (LDN) in 1995;[1] since then several other institutions have started performing LDN with the incentive to the donor having less pain, early ambulation, early resumption of regular activity, and rapid recovery.[2–6] All these studies with high levels of evidence-based results, either randomized, controlled trials or prospective, nonrandomized trials find that compared to ODN, LDN provides equal graft function, rejection rate, urological complications, and patient and graft survival. These studies, however, suggest slightly increased operative time, marginally increased warm ischemia time, and increased major complications requiring reoperation (especially in the early learning phase) in the laparoscopic cases compared to the open approach. A critical analysis of delayed graft function after LDN found that female donor kidneys into male recipients and highly HLA-mismatched donors were significant factors in delayed graft function, but that no variable related to the laparoscopic procedure itself (prolonged carbon dioxide pneumoperitoneum, warm ischemia time, renal artery length, use of right kidney) affected the functional outcome of the allograrft.[7]

Recognizing the disincentives of the ODN and understanding the advantages of the small retrieval incisions performed during LDN, few surgeons have stared performing “mini- incision” ODN. The intention of this article is to review the status of “mini-incision” ODN in comparison to LDN.

## CURRENT STATUS FOR DONOR NEPHRECTOMY: A GLOBAL SCENARIO

LDN is technically demanding and relatively new procedure compared to ODN. The number of centers in the world, which have started learning and performing LDN, is increasing day-by-day.[8–10] Even at community hospitals, LDN is considered the procedure of choice.[11] The rising number of centers performing LDN reflects that it is more accepted by the surgical community. Many centers have shown more number of living kidney donation with introduction of the laparoscopic procedure.[12] Predonor nephrectomy survey has clearly shown the preference by potential kidney donors to the LDN.[13]

### Does side matters

Initial report of laparoscopic right-sided donor nephrectomy was related with high incidence of graft loss.[14] However, with refinement of technique, many centers have performed right-sided donor nephrectomy whenever indicated. Modi *et al*., have demonstrated that how to take cuff of inferior vena cava during procurement of right renal allograft akin to ODN.[15] Further, they have shown that when multiple renal veins are present on the right side, the renal allograft could be procured laparoscopically and transplanted successfully. [16] Dols *et al*., have suggested to use right kidney routinely rather than the left kidney since right side LDN is quicker than the left side surgery and the outcome of both right side and left side donor nephrectomy are same.[17]

### Arterial multiplicity and anomalous vasculature

Transplantation with multiple renal arteries is increasing. Desai *et al*., compared outcome of kidney procured laparoscopically having multiple renal arteries and single renal artery and found no difference in the outcome.[18] Also, arterial multiplicity does not have negative impact on urological complications. Modi *et al*., have reported procurement of right-sided kidney having pre and postcaval renal artery safely by laparoscopic procedure.[19] Anomalous renal veins are also not contraindications for left-sided donor nephrectomy.[14,20]

### Obesity and laparoscopic donor nephrectomy

Obese donors require longer incision during open surgery and often the morbidity related to incision is high. Heimbach *et al*., have shown that LDN in obese donors is feasible and safe and does not result in a high rate of major perioperative complications.[21] Operative times were longer but overall length of stay was similar among obese patients. There was a 9-10% rate of wound complications in obese donors compared to 2-4% for nonobese donors.

### Laparoscopic donor nephrectomy with concomitant other surgery

Johns Hopkins group has shown the effectiveness of concomitant surgery for benign pathology at the time of performing LDN.[22] Though the operative time was more, all the procedures carried out successfully. Additional ports may be needed but additional incision for extraction of specimen is avoided.

## MINI-INCISION DONOR NEPHRECTOMY

### Evolution of mini-incision donor nephrectomy

The disincentives of ODN by standard flank approach are important reasons for development of laparoscopic surgery. As mentioned previously, the LDN is difficult to learn, and hence, few surgeons have started mini-incision ODN. Few reports of mini-open procedures have been published with smaller incisions, shorter lengths of stay and less analgesic, reflecting the ability to reduce apparent morbidity, compared to standard ODN. It is likely that these papers would have never been written if laparoscopy had not emerged on the horizon.

### Definition of mini-incision donor nephrectomy

Kok and colleague performed MIDN using an 8-15 cm (depending on body mass index) skin incision anterior to the 11^th^ intercostals space towards the umbilicus.[23] The muscles were split, ensuring preservation of underlying branches of the thoracic nerves. A vertical pararectal incision with a length of 8-10 cm was performed beginning below the costal arch by a group of surgeons from Germany.[24] Shrivastava *et al*., compared subcostal versus transcostal MIDN with or without rib resection.[25] All these approaches give upper abdominal incisions and there is no consensus regarding the site and length of such incisions. In contrast, the retrieval incision, in case of pure LDN, is usually in the lower abdomen.

### Outcome after MIDN and comparison tolaparoscopic donor nephrectomy

There are only few studies comparing laparoscopic and mini-incision approach for living donor nephrectomy. Perry *et al*., demonstrated that the MIDN is inferior to the laparoscopic approach in many domains.[26] The laparoscopic group had significantly less postoperative pain and required less time to return to normal functional activities than the mini-incision group. In addition, the laparoscopic group showed significantly higher quality of life scores than the mini-incision group.

In a prospective randomized trial comparing psychosocial and physical impairment after MIDN and LDN, Kok *et al*., have shown that the mini-incision open nephrectomy is especially associated with delayed resumption of normal activities such as return to full activity and driving, and with a diminished quality of life.[23] The probable mechanism explained was the stretching of the abdominal muscles during MIDN results in bruised muscles due to contusions and it takes several weeks to recover. When self retaining retractor blades are used, it may cause nerve stretching and compression leading to neuropraxia. When performing LDN, stretching of the rectus abdominis muscles during extraction of the kidney only lasts for few minutes. This can result in muscular pain during the first few days, but will not take weeks to recover.

Neipp *et al*., have noted a statistically significant higher incidence of ureter leak following MIDN compared to the ODN group.[27] They considered it as a price for the limited surgical access using MIDN.

There are no large series of MIDN with multiple renal arteries, anomalous renal vasculatures, and obese donors. Kok and colleagues have considered 15 cm long incision as mini-incision in their study for obese donors.[23] A mini- incision ranging up to 15 cm cannot be considered a mini- incision even in an obese donor and should be concordance with the definition of a conversion in laparoscopic surgery such as when the intended incision must be enlarged.

## DONOR NEPHRECTOMY IN INDIAN SCENARIO

In India, majority of large transplant centers perform LDN. Till June 2009, at Institute of Kidney Diseases and Research Centre, Ahmedabad, we have performed over 500 retroperitoneoscopic donor nephrectomy and over 100 LDN. At Muljibhai Patel Urological Hospital, Nadiad, 537 LDN were performed. Other centers such as Sanjay Gandhi Post Graduate Institute, Lakhnow; Christian Medical College, Vellore; All India Institute of Medical Sciences, New Delhi; and many more teaching institutions have adopted LDN as the principle mode of surgery for kidney donation. Most of these are premier teaching institutions and training the students for various laparoscopic surgeries including donor nephrectomy. This gives a background for future generation for offering LDN, both in teaching and non- teaching institutions and other centers for transplantation. ODN, though standard of care at most of these teaching institutions, is used only at rare occasions. Majority of the time ODN is performed by classical flank approach with or without rib resection. Training in MIDN is lacking even at majority of teaching institution. Only one center in India has published data of mini-incision donor nephrectomy. [25] This underscores limited acceptance of MIDN. Further, cost- effective LDN has removed the incentives for opting ODN in the developing country such as India.[28]

## CONCLUSION

LDN has evolved over a period of ten years and there are adequate data to accept it as a new standard of care for the donor. Evolution of mini-incision ODN is associated with upper abdominal incision and, randomized controlled trials with high level of evidence have suggested that LDN is having better outcome than the mini-incision ODN.
